# A comparison of the efficacy of three different peak airway pressures on intraoperative bleeding point detection in patients undergoing thyroidectomy: a randomized, controlled, clinical trial

**DOI:** 10.1186/s12893-020-00728-5

**Published:** 2020-04-10

**Authors:** Cigdem Akyol Beyoglu, Serkan Teksoz, Aylin Ozdilek, Murat Akcivan, Emre Erbabacan, Fatis Altindas, Guniz Koksal

**Affiliations:** 1 Istanbul University- Cerrahpasa Cerrahpasa School of Medicine, Department of Anaesthesiology and Reanimation, Kocamustafapasa Street, Fatih, Istanbul, Turkey; 2 Istanbul University- Cerrahpasa Cerrahpasa School of Medicine, Department of General Surgery, Kocamustafapasa Street Istanbul, Istanbul, Turkey; 3 Siirt State Hospital, Department of Anaesthesiology and Reanimation, Siirt, Turkey

**Keywords:** Intraoperative bleeding point, Peak airway pressure, Thyroidectomy

## Abstract

**Background:**

Various techniques are used to detect intraoperative bleeding points in thyroid surgery. We aimed to assess the effect of increasing peak airway pressure to 30, 40 and 50 cm H_2_O manually in detecting intraoperative bleeding points.

**Methods:**

One hundred and 34 patients scheduled for total thyroidectomy were included to this prospective randomised controlled clinical study. We randomly assigned patients to increase peak airway pressure to 30, 40 and 50 cm H_2_O manually intraoperatively just before surgical closure during hemostasis control. The primary endpoint was the rate of bleeding points detected by the surgeon during peak airway pressure increase.

**Results:**

The rate of detection of the bleeding points was higher in 50 cm H_2_O Group than the other two groups (15.9 vs 25.5 vs 40%, *P* = 0.030), after pressure administration, the HR, SpO_2_, and P peak were similar between groups (*P* = 0.125, 0.196, 0.187, respectively). The median duration of the bleeding point detection after the pressure application was 21.82 s in 30 cm H_2_O, 25 s in 40 cm H_2_O, and 22.50 s in 50 cm H_2_O groups. Postoperative subcutaneous hematomas or hemorrhages requiring surgery were not seen in any patient.

**Conclusions:**

Manually increasing peak airway pressure to 50 cm H_2_O during at least 22.50 s may be used as an alternative way to detect intraoperative bleeding points in thyroid surgery.

**Clinical trial registration:**

NCT03547648. Registered 6 June2018

## Background

Thyroidectomy has been one of the most frequently performed surgical operations in general surgery clinics in many countries. Because it is performed quite often in endemic areas for thyroid diseases, such as Turkey, hemostasis is a part of the surgical procedure that is of utmost importance. Postoperative bleeding is one of the significant complications of thyroid surgery, and it is associated with significant morbidity and mortality, increased duration and costs of surgery, and prolonged postoperative hospital stay [1].

The occurrence of a hematoma can be life-threatening due to the possible compression of the airway [2]. Therefore, strict bleeding control during thyroid surgery is vital.

To avoid postoperative bleeding a fine hemostasis and a dry surgical field are required [3]. Increasing the peak airway pressure (PAP) may help the surgeon to pinpoint the bleeding spots intraoperatively by increasing the intrathoracic and internal jugular vein pressure. Various methods are used during surgery in order to control bleeding, such as the Trendelenburg position (TP) or Valsalva Maneuvers (VM) [1, 2, 4]. Yet, to what level the intrathoracic pressure should be increased and the duration of said increase has not been standardized [2, 5].

Our aim in this study is to compare the effectiveness of three different PAP values in the detection of intraoperative bleeding points in total thyroidectomies. Our second aim is to identify the effect of bleeding point detection during surgery on postoperative hemorrhage control in total thyroidectomies.

## Methods

This is a single center, prospective, randomized, controlled study conducted in the general surgery OR at the University Hospital of Cerrahpasa Medical Faculty. Written informed consent was obtained from the study participants. Ethical approval for this study (Ethics Committee No. 172208) was provided by the Ethical Committee of Istanbul University- Cerrahpasa on 10 May 2018. The study was registered with the Clinical Trials of the US National Institutes of Health with registration number NCT03547648 on 6 June 2018 as well, and it was conducted in accordance with the Helsinki Declaration. The study was conducted with ASA I-II patients between 18 and 60 years-of-age who were undergoing a total thyroidectomy in Cerrahpasa Medical Faculty General Surgery OR. The enrollment of the patients to this study was started on 15 June 2018 and finished on 16 November 2018. Patients with lung diseases, cardiac conduction defects, glaucoma, intracranial masses and bleeding, and coagulation disorders were excluded from the study.

Patients were randomized into three groups via a computer-based randomization system (https://www.randomizer.org/).

All patients were taken to the operating room and applied standart monitoring according to ASA guidelines (ECG with 3 leads, noninvasive blood pressure, and peripheral oxygen saturation). Anesthesia was induced with propofol 2 mg kg^− 1^ (Propofol, %1, Fresenius, Fresenius Kabi, Germany) and fentanyl 2 μg kg^− 1^ (Talinat, 0.5 mg/10 ml, VEM, Turkey). Following the start of hypnosis, rocuronium 0.3–0.4 mg kg^− 1^ (Muscuron, 50 mg/5 ml, Koçak Farma, Turkey) was administered, and endotracheal intubation was performed. Anesthesia was maintained with sevoflurane 2% (Sevorane, Abbot, USA) in a 40% oxygen/air mixture in 4 L of fresh gas flow. All patients were ventilated using pressure-controlled mode (PCV) under 7 cmH_2_O PEEP with a 1:2 inspiratory to expiratory ratio, a respiratory rate between 10 and 12, a flow rate of 4 l/min, and a pressure support of 12–14 cmH_2_O to achieve EtCO_2_ values between 32 and 36 mmHg. When systolic arterial pressure (SAP) or HR increased over 20% of the initial levels, 50 μg fentanyl was added.

In all cases, the surgery was performed by the same general surgeon (S.T.) who was blinded to study groups. In the surgical unit, a sutureless thyroidectomy performed by LigaSure™ Precise LF1212 (Medtronic, USA) is the preferred technique for a total thyroidectomy. LigaSure is a bipolar dia-thermy device that uses a feedback sensor system to signal the completion of coagulation as well as to allow the termination of that signal with its special latch. Before surgical closure, the peak inspiratory pressures of the patients were increased manually to 30 cmH_2_O, 40 cmH_2_O, and 50 cmH_2_O in Group I, Group II, and Group III, respectively, with a Maquet Flow I anesthesia machine (Maquet Flow I-AGC, Rastatt, Germany), using the reservoir bag. When fresh gas flow was 4 L/min and the APL valve was set to 30, 40 and 50 cm H_2_O in accordance with the study group, the peak pressure was observed on the monitor of anesthesia device. In all groups, the pressures were kept elevated either for 30 s or upon observing the first spot of bleeding. The procedure was repeated once more for each group. All VM was applied by the same anesthesiologist (C.A.B.).

After the increase in the PAP, the number of bleeding spots, the time the spots were determined, and the size of the bleeding vein (< 2 mm or > 2 mm) were recorded. When considering the vessel size, we asked the surgeon if he used the energy-based vessel-sealing device due to a small size vessel (< 2 mm) or tied the vessel due to its larger size (> 2 mm) [1]. In addition, in all patients, the weight of the extracted thyroid gland, SpO_2_, HR, EtCO_2_, and the existence of postoperative surgical hemorrhage or hematoma were recorded. When initiating applying VM, we pressed the button to measure SAP and DAP values non- invasively during the maneuver. The measured SAP and DAP values were considered as hemodynamic parameters during PAP increase. The 1st hour SAP and DAP values, postoperative nausea-vomiting scores (PONV), and numeric rating pain score (NRS) were also recorded [6, 7].

The primary endpoint of the study was the rate of bleeding point detection after first pressure increase. The rate of bleeding point was determined as the number of patients who were detected a bleeding point compared to all patients included in that group.

Secondary endpoints were the rate of postoperative hemorrhage, intraoperative bleeding point detection time and hemodynamic parameters of patients during PAP increase.

We calculated power analysis according to the results obtained from the pilot study (*N* = 96 patients). Therefore, the first error was 5% (bidirectional), the second error was 5% (95% CI), and the bleeding frequency was 5.9% (*p* = 0.004) in the 30 cmH_2_O pressure group (Group I); following, it was 13.8% in the 40 cmH_2_O pressure group (Group II), and it was 36.4% in the 50 cm cmH_2_O pressure group (Group III). Considering the rate of bleeding point was the least in Group I (5.9%) and was the highest in Group III (36.4%), the difference of two groups was statistically significant (p = 0.004). To have at least 44 patients in each group, we decided to include a total of 150 patients, considering the probable data loss.

Normality control was done by plotting the Shapiro Wilk test, histograms, Q-Q plots, and box plot graphics. Data were given as the mean, standard deviation, median, minimum, maximum, frequency, and percentage. The normal distribution variables of the three pressure groups were compared with one-way variance analysis (one way ANOVA), and the variables that were not normally distributed were compared by Kruskal Wallis one-way variance analysis. Kruskal Wallis post multiple comparisons were performed with the Dunn test. Before and after blood pressure values were compared with dependent sample t-tests in normal distribution and with Wilcoxon tests in abnormal distribution. Nominal variables were evaluated by chi square tests and Fisher’s exact probability tests. Furthermore, pressures were compared by McNemar tests. The significance limit was set as *p* < 0.05 and as bidirectional. The analyses were performed using the NCSS 10 (Kaysville, Utah, USA) software program.

## Results

**Fig. 1:**
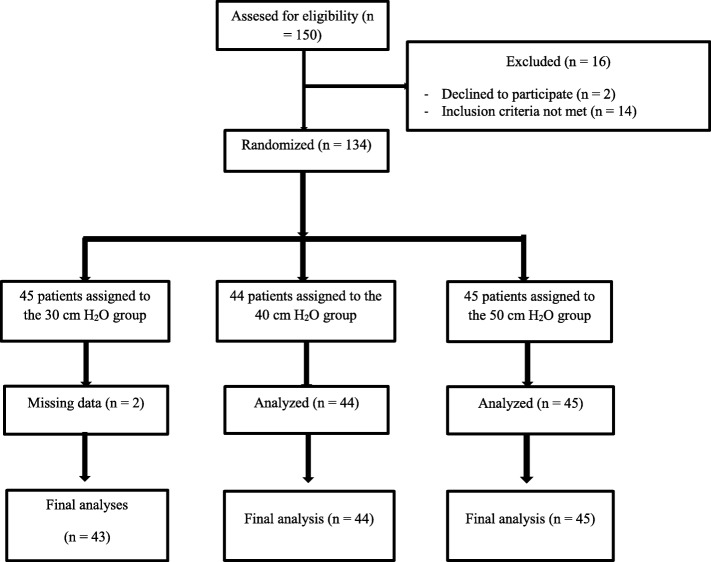
Flow diagram of the study

Between June 2018 and November 2018, 134 patients were randomized in the study (Fig. 1).

The age, ASA score, sex, height, weight, body mass index (BMI), operation time, and weight of thyroid gland were similar between the groups (Table 1).

**Table 1:** Demographic and clinical characteristics of patients in study groups

	Group I	Group II	Group III	P
Gender (F^a^/M^b^)	37/6	32/12	39/6	0.161
Age (years)	47. 07 ± 12.00	47.33 ± 14.49	51.64 ± 11.34	0.162
ASA *n* (I/II)	11/32	15/29	14/31	0.220
Height (cm)	158.64 ± 12.31	164.38 ± 10.27	161.11 ± 7.12	0.98
Weight (kg)	77.56 ± 15.71	73.02 ± 13.74	71.60 ± 13.54	0.157
BMI^c^ (kg/m^2^)	27.73 ± 6.28	27.91 ± 5.64	29.60 ± 5.16	0.236
Operation time^a^ (min)	66(40–110)	68(30–150)	68(40–110)	0.659
Thyroid weight (gr)^a^	47(10–287)	55(15–328)	64(10–800)	0.178

^a^Data are represented as Median (min-max)

a: Female, b: Male, c: Body mass index, min: minutes

The rate of detection of the bleeding points after the initial pressure was higher in Group III than the other two groups. Further, the number of detected of the bleeding focus were also similar between the groups. Moreover, bleeding point diameters were alike between the groups (Table 2).

**Table 2:** Bleeding point detection according to groups

Bleeding point detection	Group I	Group II	Group III	P
1. Pressure increase n/%	7/25.5	11/15.9	18/40^a^	0.03
2. Pressure increase n/%	1/2.2	5/11.4	2/4.4	0.17
Number of bleeding vessels *n* (1/2)	5/2	11/0	17/1	0.11
Bleeding vessel size *n* (< 2 mm/> 2 mm)	4/3	7/4	10/8	0.91

^a^Bleeding point detection is significantly higher compared to Group I and II

In Group I, bleeding was detected in 7 patients after the first pressure application, and in 1 patient after the second pressure application (*p* = 0.68), while in Group II, the bleeding focus was determined in 11 patients after the first pressure application, in 5 patients after the second pressure application (*p* = 0.06); which was not significant. In Group III, bleeding was detected in 18 patients after the first pressure application, and in 2 patients after the second application of pressure (*p* < 0.01), which was significantly different (Table 3).

**Table 3:** Comparison of the first and second pressure increase bleeding point numbers per group

Number of bleeding points	Group I	Group II	Group III
1. Pressure increase	7	11	18
2. Pressure increase	1	5	2
*P*	0.68	0.06	0.001

In Group I, SAP significantly decreased after pressure administration, but DAP remained the same. In Group II, both SAP and DAP decreased. In Group III, SAP decreased significantly, and DAP remained the same. The change of SAP and DAP were similar between groups (*p* = 0.958, *p* = 0.988 respectively). After pressure administration, the HR and SpO_2_ were similar between groups (*p* = 0.125, *p* = 0.196 respectively). In Group I, the EtCO_2_ level was higher compared with the other two groups (*p* < 0.001) (Table 4).

**Table 4:** Hemodynamic parameters of patients during peak airway pressure increases per group

Hemodynamic parameters	Group I	Group II	Group III	P
SAP	111(82–160)	111(67–163)	112(70–180)	0.958
DAP	73(47–100)	72(40–103)	73(50–100)	0.988
HR^a^	77.56 ± 15.71	73.02 ± 13.74	71.60 ± 13.54	0.125
EtCO_2_	25–42^b^	22–37	25–35	0.001
SpO_2_	97–100	96–100	95–100	0.196
Ppeak	16–43	12–49	14–55	0.187

Data are represented as Median (min-max)

^a^Presented as ‘Mean ± SD’

^bEtCO^_2_^value is significantly different in Group I compared to Group II^

The median duration of the bleeding point detection after the initial pressure application was 21.82 s in Group I, 25 s in Group II, and 22.50 s in Group III. After the second pressure application, the median duration of the bleeding point detection was 20 s in Group I, 24 s in Group II, and 25 s in Group III. Additionally, the postoperative SAP and DAP values were similar between groups (*p* = 0.446, *p* = 0.234 respectively) (Table 5). Furthermore, PONV and postoperative NRS scores were similar between the groups (*p* = 0.87, *p* = 0.63 respectively) (Table 6). Finally, postoperative subcutaneous hematomas or hemorrhages requiring surgery were not seen in any patient.

**Table 5:** Postoperative systolic and diastolic arterial pressure values according to groups

	Group I	Group II	Group III	P
SAP^a^	135(104–166)	131(108–165)	132(110–177)	0.446
DAP^b^	78(55–98)	78(53–103)	75(60–92)	0.234

Data are represented as Median (min-max)

a: Systolic arterial pressure

b: Diastolic arterial pressure

**Table 6:** Postoperative numeric rating and vomiting scale scores according to groups

Patients NRS and PONV scores	Group I	Group II	Group III	P
NRS^a^ n(0/1/2/3/4)	20/1/17/3/2	26/2/13/2/1	21/4/15/3/2	0.87
PONV^b^ n(0/1/2)	42/1	42/1/1	41/3/1	0.63

a: Numeric rating scale, b: Postoperative nausea and vomiting scale

## Discussion

The efficacies of the three different PAP for bleeding point detection in total thyroidectomies were compared for the first time in this study. The 50 cmH2O peak pressure increase was significantly superior in the detection of bleeding points.

Postoperative bleeding prevention is especially important in head and neck surgery because it may cause fatal complications. Drains are not included in the routine practice of the present study’s hospital, because the use of a drain after thyroid surgery increases postoperative pain and the length of hospital stay with no decrease in reoperation rate, hematomas, or seroma formation [3, 8–10].

The VM, which is frequently applied for bleeding focus control, is known to cause a backflow of venous blood by increasing the internal jugular vein pressure [11]. Jungieira [12] stated that the VM should be performed for at least 15–20 s with 40 cmH_2_O intraoral pressure to be effective. He emphasized that the effectiveness of VM should also be confirmed by monitoring hemodynamic responses. However, the baroreceptor and chemoreceptor reflex responses of patients under general anesthesia are suppressed, and a standard method of intraoperative VM has not been defined [2, 13, 14].

Therefore, there are differences in the use of VM for intraoperative bleeding control. In neurosurgical operations, it has been shown that the application of 40 cmH_2_O peak pressure with the reservoir bag of the breathing circuit for 10 s can be effectively used for the control of venous hemostasis [4, 15]. In a similar application, the treatment of patients with 30 cmH_2_O PEEP in thyroid surgery has been shown to determine intraoperative bleeding foci in 32% of patients [5]. In the present study, we identified the bleeding points in 40% of patients with 50 cmH2O PAP. This rate was significantly higher than the other groups. Therefore, as a result of our study, it can be said that 50 cmH_2_O peak pressure can be used more effectively than other pressure levels in detecting bleeding points in total thyroidectomies. Another important point concerning the 50 cmH_2_O group was that the rate of detection of bleeding was significantly lower in the second airway pressure increase compared with other groups. This situation shows us that 50 cmH_2_O pressure can be used effectively in the detection of bleeding foci in the first application.

In general surgeons are willing to increase PAP as much as high levels -at least 40 cm H_2_O pressure- to feel confident during surgical closure. Besides, anesthesiologists hesitate to increase PAP to 50 cmH_2_O in the belief that PAP increase will cause barotrauma. However, increased PAP-related barotrauma is not as frequent as supposed; transmural pressure is the main factor causing barotrauma [16]. Increased driving pressure is the determined main factor causing lung injury after surgery [17]. Hence, applying recruitment maneuvers with high PAP for 20 to 30 s during surgery has been approved to avoid atelectasis [17]. In a previous study [18] researchers declared a negative correlation between a higher PAP and barotrauma. Anesthesiologists should feel comfortable to increase PAP to 50 cm H_2_O pressure as a part of patient safety avoiding postoperative life-threating hematoma. Thus, it seems that avoiding high PAP in patients who have increased intracranial or intraocular pressure is more meaningful. Nevertheless our study did not include elderly patients with lung diseases and cardiac conduction defects, so we cannot claim that increasing PAP to 50 cmH_2_O may not cause barotrauma or cardiac complications in these patients.

Presently, there is no consensus concerning the duration of VM that must be applied in the control of intraoperative hemostasis. In previous studies, it has been reported that VM should be applied in 30–60 s, 15–20 s, or 20–40 s periods [2, 4, 5, 15, 19]. In the present study, we found the mean duration of the detection time of the bleeding points in Group I, II and III as 21.82, 25 and 22.50 s respectively. Therefore, as a result of our study, we can say that maneuvers applied in total thyroidectomies during intraoperative hemostasis control should be applied for at least 20–25 s, similar to the results of previous studies.

The effects of VM on hemodynamic parameters were examined in detail in previous studies [20–23]. It has been observed that arterial hypotension, reflex tachycardia, hypertension, and reflex bradycardia responses generally develop, respectively, in awake patients [2]. However, in our study we did not see a significant change in HR during PAP increase, which may be explained by the dulling or delaying effect of general anesthesia on baroreceptor reflex response [13, 14, 24]. Abnormal reflex response to VM under general anesthesia was shown in previous studies [4, 25, 26].

In our study, no hemorrhages or hematomas were observed in any patient in the postoperative period. Although there is no consensus on the risk factors contributing to postoperative hemorrhages in thyroid surgery, postoperative hypertension (HT), nausea and vomiting, the length of surgery, and the extent of the surgery performed are the most likely risk factors leading to a postoperative hematoma or hemorrhage [27–29]. Among these factors, HT was considered to be the most risky one [27]. In the daily routine practice of the XXX Cerrahpasa Medical Faculty General Surgery department, we admit all patients in normotensive ranges that undergo a total thyroidectomy. Also, surgeons use energy-based vascular coagulation techniques to prevent the risk of postoperative complications [3]. In our study, during the postoperative period, treatment-resistant nausea and vomiting were not observed in any patient. In addition, the duration of operation was 150 min in only one patient in Group II while the mean duration of operation was 110 min for the remaining patients. We posit that all patients were free of postoperative bleeding due to the precautions we took to prevent postoperative hemorrhage. Even so, we have to declare that increasing PAP any of three different airway pressures did not show an effect on postoperative hemorrhage. We believe this issue needs to be further evaluation.

There are several ways to provide hemostasis control during thyroid surgery, such as TP or abdominal compression. However, TP may cause a significant increase in intracranial and intraocular pressure, restricting the use of this technique in the detection of bleeding points [5]. Abdominal compression added to TP may cause a similar effect. Another possible limitation to the use of TP is that the displacement of the endotracheal tube during TP may cause a challenge for anesthesiologists due to the difficulty of accessing the patients under the sterile covers in thyroid surgery.

The rate of postoperative hemorrhage after thyroid surgery is stated as 1.47% [30]. However this ratio seems to be lower, hemorrhage may cause fatal complications. In our study we could not prove a relation between intraoperative bleeding and postoperative hemorrhage. In order to evaluate postoperative bleeding after a thyroidectomy, a new study design targeting primary aim of postoperative hemorrhages should be planned, in which more patients should likely be included. We can say that this is a limitation of our study. Another limitation was that there is not a control group in our study. A control group might be useful to show the effectiveness of intraoperative VM on prediction of postoperative hemorrhage. Hence, VM applied with each 30, 40 and 50 cm H_2_O PAP will help identify the intraoperative bleeding points and prevent postoperative hemorrhage. Another limitation of our study is that our study population included ASA I-II patients aged between 18 and 60 years. However, patients undergoing thyroid surgery may be elderly and may have cardiac and pulmonary comorbidities; so, the study results may not be applicable to the all thyroid patients.

## Conclusion

In the determination of intraoperative bleeding points, keeping airway pressure at 50 cm H_2_O for 22.5 s may be more effective in patients undergoing total thyroidectomies, as compared with 30 and 40 cm H_2_O pressures.

However, we cannot assert that any PAP is superior to another on preventing postoperative hemorrhage. The effect of detecting bleeding spots intraoperatively on postoperative bleeding control was determined by this method and is a subject for further investigation in more comprehensive, randomized clinical studies.

## Data Availability

The datasets used and/or analysed during the current study are available from the corresponding author on reasonable request.

## Abbreviations

TP: Trendelenburg position
VM: Valsalva maneuvers
PAP: Positive airway pressure
SAP: Systolic arterial pressure
DAP: Diastolic arterial pressure
HR: Heart rate
PONV: Postoperative nausea-vomiting scores
NRS: Numeric rating pain score
BMI: Body mass index
